# Untying the Knot: A Rare Case of Formation of a Life-Threatening Intracardiac Knot Following the Placement of a Temporary Transvenous Pacemaker

**DOI:** 10.7759/cureus.33188

**Published:** 2022-12-31

**Authors:** Esiemoghie J Akhigbe, Mohamed Suliman, Rameez Sayyed

**Affiliations:** 1 Internal Medicine, Marshall University Joan C. Edwards School of Medicine, Huntington, USA; 2 Cardiology, Marshall University Joan C. Edwards School of Medicine, Huntington, USA

**Keywords:** pacemaker lead repositioning, 3rd degree heart block, pacemaker leads, pacemaker extraction, pacemaker complication

## Abstract

The implantation of a temporary pacemaker lead is a very common procedure performed in most hospitals and is known to be relatively safe, but there can be serious complications in rare circumstances. Reported complications including arrhythmias, infection, thromboembolic phenomena, and perforation of the vessel or the heart are all extensively described. However, an unusual and life-threatening complication that is not frequently discussed is the formation of intracardiac knots. We present a case of a rare complication of a temporary pacemaker placement with the formation of a knot in the distal lead requiring expert technique for removal.

## Introduction

Since the invention of temporary transvenous pacemakers (tTPM), their use for the acute management of bradyarrhythmia has been on the rise. Although useful in the medical management of arrhythmias, it had been plagued with multiple complications that include arrhythmias, infection, thromboembolic phenomena, and perforation of the vessel or the heart [1]. However, distal lead knotting following the placement of a tTPM has not been extensively described. Intracardiac knotting could lead to vascular or valvular injury, pneumothorax, symptomatic loss of pacing or hemodynamic compromise, and difficult lead removal [2].

We present the case of a 59-year-old female with bradyarrhythmia with loss of pacing following the tTPM placement who was found to have an unusual knot formed at the distal part of the lead. This case highlights an unusual and potentially life-threatening complication of the tTPM placement.

## Case presentation

**Figure 1:**
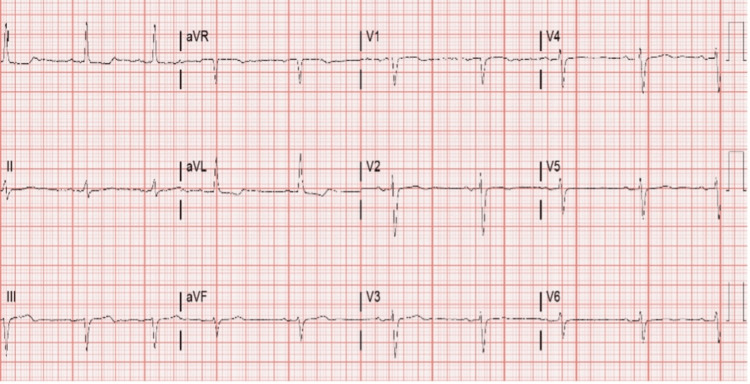
Electrocardiogram showing the junctional rhythm

A 59-year-old female presented to the emergency department after a fall from a standing height. She had a past medical history of end-stage renal disease that required hemodialysis for three years as well as paroxysmal atrial fibrillation, which was treated with Flecainide and Eliquis. Her last echocardiogram showed a moderately dilated left atrium with a normal ejection fraction. She also had a history of deep venous thrombosis as well as nonobstructive coronary artery disease. The trauma workup was negative. She did not lose consciousness or sustain any injuries. She had missed dialysis the day prior. Initial vitals showed a blood pressure of 76/36 mmHg with a heart rate of 31 bpm. Electrocardiogram revealed a junctional rhythm. Laboratory abnormalities revealed a potassium of 6.6. Preparation for urgent dialysis was made, and cardiology was consulted. However, the patient's heart rate did not improve due to a junctional rhythm with a concern for a high-grade AV block (Figure 1).

**Figure 2:**
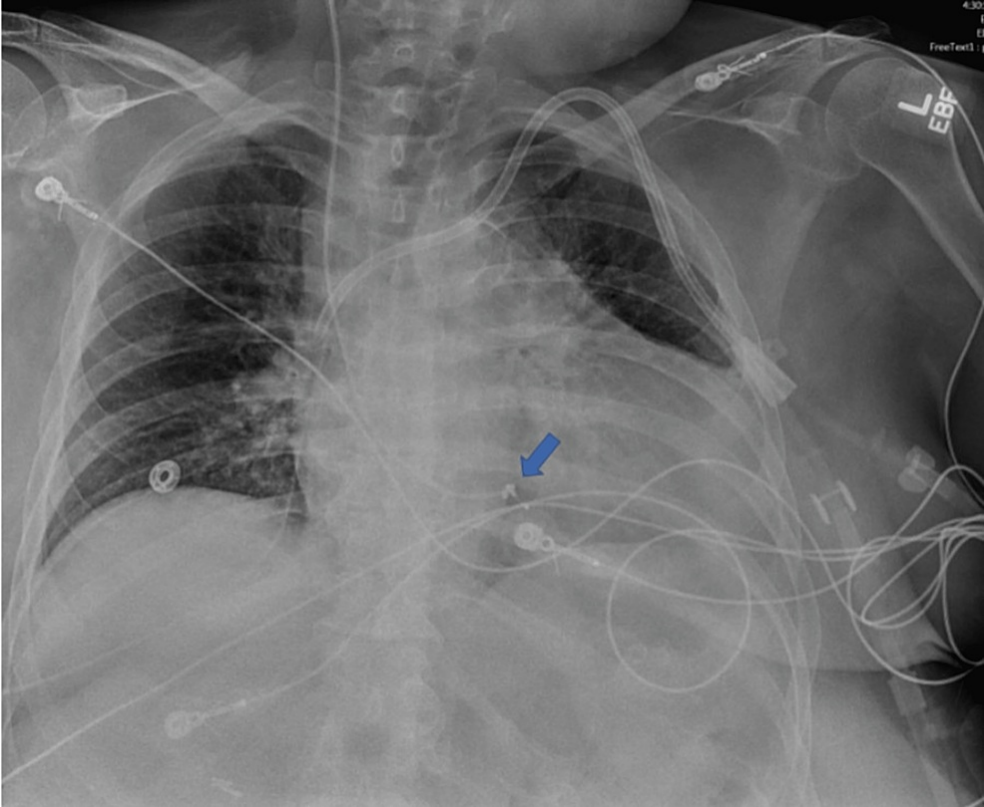
A chest radiograph with an arrow showing a distal knot in the pacemaker lead The image shows an anteroposterior radiograph with a left subclavian vas-cath. The right internal jugular temporary transcutaneous pacemaker with a distal knot is highlighted by the blue arrow.

A right internal jugular vein transvenous pacemaker was placed. Post-procedure chest x-ray revealed a temporary venous pacer tip overlying the right ventricle with redundancy at the tip (Figure 2). Initial capture was appropriate but was shortly lost, and the temporary wire was retracted and planned to be replaced; however, there was resistance upon removal. Vascular surgery was consulted, and a buddy guidewire was placed through the sheath with the removal of the sheath for protection of access; subsequently, the transvenous pacing wire was manipulated and removed revealing a knot in the distal tip. The patient's rhythm subsequently improved after consecutive dialysis sessions, and she was discharged from the hospital to a skilled nursing facility.

## Discussion

In the late 19th century, the initial descriptions of a pulsed electrical stimulation to the heart were first postulated by J. A. McWilliam. He described the pacing of the ventricles using a flexible wire electrode. The continuous evolution in the technology led to the first pacemaker device built by an American scientist named Albert Hyman in 1932. In the late 1950s, two scientists named Seymour Furman and John Schwedel were able to innovate a novel technique whereby endocardial stimulation was provided through a lead inserted via the internal jugular vein [3]. Since its innovation, temporary pacing has become a commonly performed procedure in most hospitals, with indications for its use well established by governing National Cardiology Colleges [4].

Pacemakers work by electrically stimulating the myocardium, thereby increasing the heart rate in bradyarrhythmia, or, in some special cases, it is used to prevent or treat tachyarrhythmias as seen in circuit entraining in atrial flutter and ventricular tachycardia. Temporary pacing is usually preferred in an acute situation due to its ease of placement and availability [3]. In pathologies where there is a temporary disruption in the electrical conduction of the heart, tTPM serves as a bridge to a permanent device. However, it should be known that the time of recovery could be lengthy in certain neuromuscular conditions, thereby leading to a prolonged duration of device placement [5].

Numerous complications associated with its placement have been described in the literature; they have been generally classified as (1) complications in establishing access, (2) complications of the catheterization procedure, and (3) complications of the catheter residence [6,7]. In a study conducted in 1983 with 1022 patients, there were no reported deaths, and the study reported a complication rate of only 13.7% with the pericardial rub being the most frequent (5.3%). In contrast, a study performed by Murphy involving 194 patients showed staggering life-threatening complications in 68 patients (35%) and an unusually high number of deaths in 55 patients, which accounted for 28% of the study population [7]. This high rate of complications and death was likely attributed to poor techniques and also the performance of the procedure by poorly skilled personnel [4].

According to the literature, the formation of an intracardiac knot has been widely attributed to poor technique usually as a result of operator inexperience [7] and the increased flexibility of the pacemaker lead. Although the flexibility of the lead is considered to be advantageous in the insertion of the pacemaker, it has been shown to be hazardous since its redundant lead may form into loops [1]. The use of guide wires is usually the first line and preferred technique when an intracardiac knot occurs; this technique is performed by gradually advancing the guidewire into the catheter until the knot is untied. However, this technique has its limitations as its success is dependent on the looseness of the knot. If the intracardiac knot is not loose enough and/or is located a long distance away from the tip of the catheter, it is usually unsuccessful. Another method that has been described involves traction and removal of the catheter through the puncture site. This technique requires both internal jugular and subclavian veins access, thereby increasing the risk for major local complications. Safer techniques involving combined radiological-percutaneous techniques of extraction have been described, especially if the above-mentioned technique for the untying of the knotted catheter fails. The commonly used percutaneous removal method requires the replacement of the original sheath used for catheter insertion by an introducer of a larger diameter close to that of the knot [8].

Another method, though not widely used, is to untie the knot by holding its distal end with a snare and tugging it back and forth while simultaneously holding its proximal end [9]. Basket retrieval and the use of endomyocardial biopsy forceps are two other methods described [6,9]. If the knot becomes fixated to the myocardium, is too large in size, or has multiple loops, then surgical removal is usually recommended for safer retrieval.

As with the complication discussed in this case report, this complication is easily prevented when the catheter or pacemaker is placed under fluoroscopic guidance. This allows the operator to ensure that a great length of the catheter is not introduced into the cardiac chamber, thus preventing the catheter from doubling on itself and forming a knot [1].

## Conclusions

Although the placement of tTPM is generally considered a safe procedure, knotting of the lead tip as shown in this case should be anticipated, especially following the loss of capture after the placement of a TPM. This can be easily prevented by ensuring that the length of the electrode introduced into the cardiac chamber is not too long, thereby preventing a loop formation and potential knot formation.
